# Efficacy and Safety of Three Cycles of TIP and Sequential High Dose Chemotherapy in Patients with Testicular Non-Seminomatous Germ Cell Tumors

**DOI:** 10.3390/jcm14010131

**Published:** 2024-12-29

**Authors:** Musa Baris Aykan, Gulsema Yildiran Keskin, İsmail Erturk, Ramazan Acar, Ahmet Fatih Kose, Nuri Karadurmus

**Affiliations:** 1 Department of Medical Oncology, University of Health Sciences, Gulhane School of Medicine, Ankara 06018, Turkey; gulsemayildiran@gmail.com (G.Y.K.); ierturk@hotmail.com (İ.E.); dr_racar@yahoo.com (R.A.); drnkaradurmus@yahoo.com (N.K.); 2 Department of Internal Medicine, University of Health Sciences, Gulhane School of Medicine, Ankara 06018, Turkey; afkose96@gmail.com

**Keywords:** germ cell tumors, high dose chemotherapy, salvage therapy

## Abstract

**Background**: Salvage treatment options have not been validated in relapsed or refractory germ cell tumors. Moreover, the study populations including these patients have different heterogeneities. This study aimed to evaluate the efficacy and safety of three cycles of TIP sequential high-dose chemotherapy in patients with testicular non-seminomatous germ cell tumors who relapsed or had a refractory course after first-line platinum-based chemotherapy. **Methods**: Data of 141 patients who underwent three cycles of TIP followed by HDCT due to relapsed/refractory gonadal NSGCTs after first-line cisplatin-based chemotherapy (BEP/EP) at Gulhane School of Medicine Hospital Medical Oncology Department between January 2017 and May 2024 were evaluated retrospectively. Patients underwent a treatment regimen consisting of two phases. Initially, they received three cycles of induction therapy using a combination known as TIP, which includes paclitaxel, ifosfomide, and cisplatin. Following this, they were given a single cycle of high-dose chemotherapy. Demographic and clinicopathological features of patients and treatment-related complications and survival outcomes were recorded. **Results**: Median follow-up for all patients was 35.2 (95% CI, 29.45 to 41.07) months. Complete Response (CR) or marker negative Partial Response (PR) after HDCT was achieved in 84 (59.6%) patients. Median time for PFS not reached (NR) (95% CI, NR) in the entire group. The 2-year PFS rate was 51.8%. Median time for OS not reached (95% CI, NR) and the 2-year OS rate was 72.3%. The most common myelotoxicity observed after HDCT until engraftment was grade 4 neutropenia (100%) and grade 4 thrombocytopenia (96.5%). Transplantation-related mortality occurred in 7.1% of patients. Variables that remained statistically significant in multivariable analysis and were associated with poor prognosis for overall survival were platinum refractory disease and AFP and/or beta HCG elevation. **Conclusions**: Significant survival can be achieved after three cycles of TIP consecutive HDCT, while treatment-related mortality was found to be low.

## 1. Introduction

Germ cell tumors (GCTs) are curable even in the setting of relapsed or refractory disease, but treatment still remains challenging [1]. Approximately one-quarter of patients initially treated with cisplatin-based combination therapy will require a new treatment approach due to relapse or refractory disease [2]. But cure or long-term remission can still be achieved with salvage treatments. The currently preferred treatments for second line and beyond are conventional dose chemotherapy (CDCT) or high-dose chemotherapy (HDCT). Treatment guidelines suggest that there is no clear superiority of CDCT or HDCT over each other [3].

In the selection of CDCT, several combinations stand out with high response rates. Response rates are 50–60% by using vinblastine (VeIP) or paclitaxel (TIP) in addition to ifosfamide and cisplatin [3]. However, when HDCT is used with direct peripheral autologous stem cell transplantation (ASCT) in salvage, recovery rates appear to be superior [3]. But the fact that HDCT should be performed in experienced centers with high patient volume is also one of the limiting factors in treatment [4]. Because phase III randomized trials are difficult to conduct in uncommon cancers such as relapsed or refractory GCTs, treatment decisions are often based on results from retrospective studies. Most phase III HDCT trials have shown no survival benefit [3]. But these prospective studies have a small number of patients and some have been terminated early due to toxicity [3]. These studies substantially included seminoma and non-seminomatous GCTs (NSGCTs). Moreover, patients with extragonadal GCTs were also included. Therefore, different responses may be obtained to the treatments applied due to the disease biology. In addition, HDCT can be used once a year in countries with resource limited countries so the generalizability of the existing data to the whole world is limited. The number of studies evaluating patients who underwent HDCT without waiting for a second recurrence after CDCT is at an anecdotal level [5].

In daily practice, histological, clinical and biochemical parameters that try to define prognosis play a role in making treatment choices. Recent studies continue to show that the tumor microenvironment and inflammation in this area are important in many stages of cancer pathogenesis, especially invasion and angiogenesis [6]. The systemic immune inflammation index (SII) is calculated by the number of lymphocytes, neutrophils and platelets in peripheral blood. SII has been studied in various tumors with the idea that it can indicate inflammation in the tumor microenvironment and be a predictor of treatment response and has been reported to be an important biomarker for GCTs [6].

We aimed to evaluate patients who were treated with three cycles of TIP for induction only in gonadal NSGCTs male patients and then HDCT without waiting for a new recurrence. We aimed to evaluate the survival characteristics and side effects profile seen after HDCT in this highly selected group. We also aimed to examine the SII in terms of its relationship with progression-free survival (PFS) and overall survival (OS).

## 2. Materials and Methods

Data of 141 patients who underwent three cycles of TIP followed by HDCT and ASCT due to relapsed/refractory gonadal NSGCTs after first-line cisplatin-based chemotherapy (BEP/EP) at Gulhane School of Medicine Hospital Medical Oncology Department between January 2017 and May 2024 were evaluated retrospectively. All patients were over 18 years at the time of HDCT. Patients who had radiological or biochemical progression after first-line standard cisplatin-based therapy for advanced disease and received three cycles of TIP followed by HDCT in sequential therapy were included. Patients who were lost to follow-up, had insufficient follow-up time, or had missing data were excluded from the study. Platinum-refractory disease is characterized by the progression of the disease occurring within four weeks after the last administration of cisplatin in first-line treatment. All patients received three cycles of TIP (paclitaxel 250 mg/m^2^/day D1, ifosfamide and mesna 1500 mg/m^2^/day D2–D5 and cisplatin 25 mg/m^2^/day D2–D5 every 21 days) as induction therapy before HDCT. The procedure for harvesting CD34+ stem cells involved administering 10 mcg/kg of granulocyte colony-stimulating factor (G-CSF) via subcutaneous injection for five days, starting one month after the third cycle of the TIP. The patients underwent stem cell harvesting before HDCT. The HDCT protocol, known as the CE regimen, included the carboplatin and etoposide which given at a dosage of 700 mg/m^2^ per day over three consecutive days (days -5, -4, and -3) and 750 mg/m^2^ per day for the same three days, respectively. The stem cell transplant was conducted on day 0, following the completion of the HDCT. In patients who underwent the ICE protocol as HDCT, the total dose was 12 g/m^2^ ifosfamide and mesna, 1200 mg/m^2^ carboplatin and 1200 mg/m^2^ etoposide divided into six days (days -8, -7, -6, -5, -4, -3 and followed by stem cell transplantation on day 0. Pre-HDCT harvested autologous stem cells (at least 2.5 million CD34+ cells per kilogram of body weight) were reinfused into patients two days after the last chemotherapy. GCSF therapy was then started the following day. All patients were administered antibiotics (levofloxacin), antivirals (valacyclovir), and antifungals (oral fluconazole) as a preventative measure against infections. In addition, prophylactic antiemetics were given to manage nausea and vomiting, and oral care products were regularly included in the treatment protocol to address mucositis. Patients underwent daily monitoring of blood counts, biochemistry panels, C-reactive protein (CRP), and procalcitonin levels until engraftment. Platelet engraftment was defined as a platelet count exceeding 20,000/mm^3^ for three consecutive days. Similarly, neutrophil engraftment was defined as an absolute neutrophil count of 2000/mm^3^ or greater. Platelet and erythrocyte transfusions were administered as needed to maintain platelet counts above 10,000/mm^3^ and hemoglobin levels above 8 g/dL, respectively. This study retrospectively reviewed patient data, including demographic information, clinicopathological characteristics, and International Germ Cell Cancer Collaborative Group (IGCCCG) risk group assignment at diagnosis. Pre-HDCT serum alpha-fetoprotein (AFP) and beta human chorionic gonadotropin (HCG) levels were also collected. Treatment-related complications, PFS and OS were analyzed using patient records and hospital software systems. The classification of adverse events was based on the NCI Common Terminology Criteria (NCI CTCAE v5.0). SII was calculated as platelet count (cells/mm^3^) × neutrophil count (cells/mm^3^)/lymphocyte count (cells/mm^3^) obtained from hemogram one month before HDCT. Transplantation-related mortality (TRM) was evaluated as death from any cause occurring within the first 100 days after HDCT. Radiological response was assessed by positron emission tomography/computed tomography (PET/CT) or conventional CT three months after treatment. Tumor marker levels were also examined during this assessment. This study was approved by the Health Sciences University Gulhane Scientific Research Ethics Committee, Ankara, on 8 October 2024, with approval number 2024/7451. IBM SPSS v. 25 program was used in all statistical procedures. We conducted a descriptive analysis of patient characteristics, reporting frequencies and percentages for categorical variables. The cut-off value for SII was determined by the receiver operating characteristic (ROC) analysis. According to this cut-off value, patients were divided into SII-high and SII-low groups according to their SII values before HDCT. Median follow-up time was determined using the reverse Kaplan–Meier method. Kaplan–Meier analysis was employed to estimate OS and PFS, with time zero defined as the first day of HDCT. The log-rank test was used to compare survival curves between groups. To identify independent predictors of survival, we performed a multivariable Cox regression analysis, including variables with a *p*-value < 0.05 in univariate analyses. Statistical significance was set at *p* < 0.05.

## 3. Results

A total of 141 patients were included in the analysis. All patients were male and had testicular NSGCTs. While the median age was determined as 27 (18–51), 61% of the patients were determined to be under 30. Mixed germ cell histology was present in 76.6% of the patients. Stage III disease was observed in 64.5%. In addition, 83 patients (58.9%) had IGCCCG poor risk features at metastatic stage. In first-line systemic therapy, 97.9% of patients received BEP or EP. At least one of the liver-bone-brain metastases was detected in 49 patients (34.8%) before HDCT. Elevated AFP and/or beta HCG levels were detected in 58 (41.1%) patients. Six patients (4.3%) had platinum refractory disease after first line treatment. Before second line treatment, IPFSG intermediate status was detected in 74 (52.5%) of the patients. CE protocol was mostly used as HDCT (95.7%).

**Figure 1:**
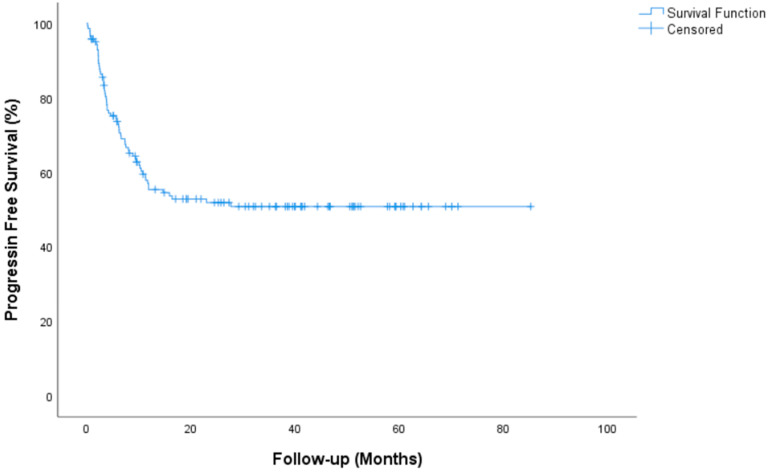
Progression-free survival graph.

**Figure 2:**
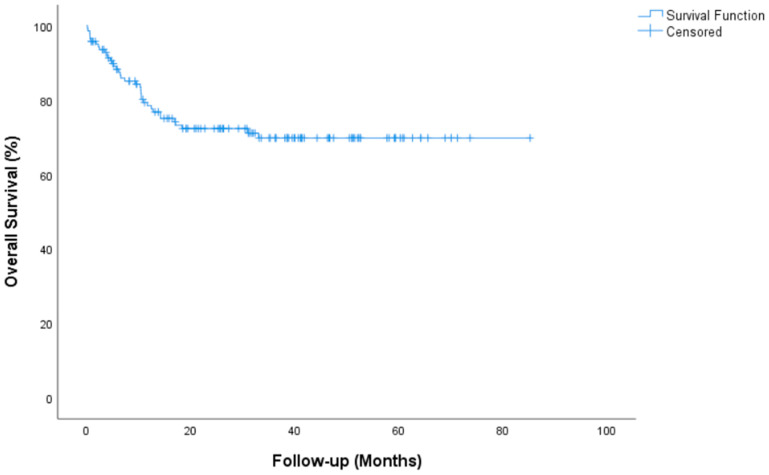
Overall survival graph.

**Figure 3:**
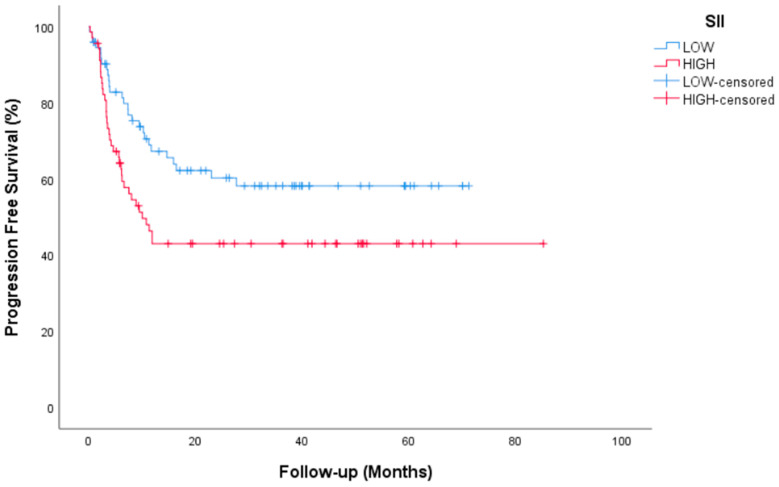
PFS by SII status.

**Figure 4:**
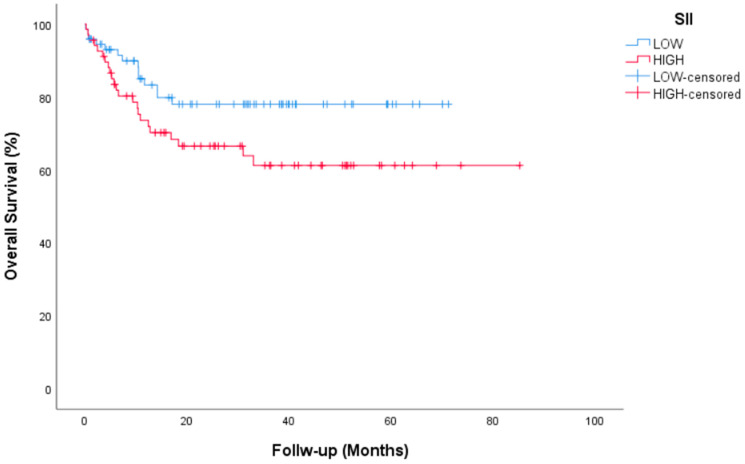
OS by SII status.

Median follow-up for all patients was 35.2 (95% CI, 29.45 to 41.07) months. Complete Response (CR) or marker negative Partial Response (PR) after HDCT was achieved in 84 (59.6%) patients. Median time for PFS was not reached (NR) (95% CI, NR) in the entire group. The 2-year PFS rate was 51.8%. Median time for OS not reached (95% CI, NR) and the 2-year OS rate was 72.3%. During the median follow-up, 104 of the 141 patients were still alive. Tumor resection surgery was performed after HDCT in 20 patients (14.2%). PFS and OS survival graphs are presented in Figure 1 and Figure 2. PFS and OS graphs according to SII being low or high are shown in Figure 3 and Figure 4.

The optimal SII threshold value was calculated as 610.18 in Receiver operating characteristic (ROC) analyses. This SII value had 59.5% sensitivity and 59.7% specificity for 2-year PFS prediction. (AUC 0.642; 95% CI 0.551–0.733; *p* = 0.004). According to this calculation, 68 of the patients (48.2%) were detected as SII high.

The most common myelotoxicity observed after HDCT until engraftment was grade 4 neutropenia (100%) and grade 4 thrombocytopenia (96.5%). Kidney failure requiring dialysis after HDCT was seen in 2.1% of patients. TRM occurred in 7.1% of patients. The baseline characteristics and subsequent HDCT follow-up-related characteristics are presented in Table 1 and Table 2.

Univariate analyses of PFS showed significance in terms of platinum refractory disease, AFP and/or beta HCG elevation and SII. Patients with platinum-sensitive disease had a median PFS of NR vs. 3 (95% CI, 2.4–3.5) months for platinum-refractory disease (*p* = 0.001). Patients with AFP and/or beta HCG elevation had a median PFS of 6.23 (3.3–9) months) vs. NR for non-elevation disease (*p* = 0.001). In terms of SII, high level patients had a median PFS of 10 (95% CI, 5.9–14) months vs NR with low level patients (*p* = 0.026). A significant difference were found for PFS in multivariate analyses between patients with platinum-sensitive and patients with platinum-resistant disease (*p* = 0.021, 95% CI, 1.22–11.2 months), between patients with AFP and/or beta HCG elevation and non-elevation patients (*p* = 0.001, 95% CI, 2.9–9.6 months) and between patients with SII high and low (*p* = 0.027, 95% CI, 1–3.2 months). Histological subtypes, IGCCCG, pulmonary metastasis, liver-bone-brain metastasis, IPFSG and showed significance in univariate analyses but lost significance in multivariate analyses.

Univariate analysis of OS showed significance in terms of platinum refractory disease and AFP and/or beta HCG elevation. Patients with platinum-sensitive disease had a median OS of NR vs. 6.56 (95% CI, 3.7–9.4) months for platinum-refractory disease (*p* = 0.001). Patients with AFP and/or beta HCG elevation had a median OS of 6.23 (3.3–9) months vs. NR for non-elevation disease (*p* = 0.001). A significant difference was found for OS in multivariate analyses between patients with platinum-sensitive and patients with platinum-resistant disease (*p* = 0.003, 95% CI, 1.14–13.21 months), between patients with AFP and/or beta HCG elevation and non-elevation patients (*p* = 0.001, 95% CI, 2.36–11.84 months). Histological subtypes IGCCCG, pulmonary metastasis, liver-bone-brain metastasis and IPFSG showed significance in univariate analysis but not in multivariate analysis. The parameters evaluated for univariate and multivariable analysis of PFS and OS are presented in Table 3 and Table 4. PFS and OS curves for all patients are presented in Figure 1 and Figure 2.

## 4. Discussion

Deciding on the option of salvage therapy for relapsed or refractory GCTs is much more complex than deciding on first-line therapy. Reasons include unclear validation of treatment options, rarity of the disease, and large heterogeneity of patient groups in reported studies. In this study, only the data of 144 patients with testicular NSGCTs who received HDCT treatment after three cycles of TIP for the first relapse/refractory disease were evaluated. In most of the studies conducted for GCTs, which constitute a relatively rare group among all malignancies, both gonadal seminoma cases and extragonadal GCTs were included in addition to gonadal NSGCTs, and some studies even included ovarian GCTs [7,8]. In this sense, a more homogeneous patient group was evaluated in our study. On the other hand, there is no clear consensus yet on whether salvage treatment should be performed with CDCT or directly with HDCT. Since HDCT cannot be performed consecutively in our country due to reimbursement conditions, consolidation with HDCT after induction with CDCT is a treatment option that we are focusing on in our center. In our approximately 3-year follow-up study, the median PFS and OS rates were not yet reached. However, we were able to determine the 2-year PFS and OS rates, which were 51.8% and 72.3%, respectively. A history of platinum refractory disease and tumor marker elevation before HDCT continued to indicate poor prognosis, as previously associated with previous studies [7,8,9]. In the IT-94 trial, which was conducted with a similar design in the past, CE plus cyclophosphamide treatment used as HDCT after three cycles of VIP or VeIP could not be clearly evaluated due to toxicity [10]. In our study, it is important to present survival data compatible with salvage sequential HDCT in the literature, along with the absence of unmanageable toxicity. In sub-analyses, the significant findings of platinum refractory disease and tumor marker elevation before HDCT in terms of both PFS and OS are important in terms of predicting poor prognosis. There is very little data on systemic inflammatory markers for GCTs. In particular, the number of studies evaluating their relationship with HDCT is rare. Here, the relationship of SII with PFS was shown, but not with OS.

In a landmark study on HDCT, Einhorn et al. evaluated the data of 184 relapsed and refractory patients with metastatic testicular cancer [11]. Most of the patients were treated with one or two cycles of standard dose vinblastine plus ifosfamide plus cisplatin followed by two consecutive 3-day cycles of high-dose CE. The 4-year disease-free survival (DFS) was reported as 63%, which is better than our study [11]. In this study, there were approximately 20% seminoma patients. The IGCCCG poor-risk patient rate in the study was approximately 20% lower than in our study. Late relapse patients were not included in this study, but they were present in our study at a rate of 15%. In addition, the fact that a large portion of the patients in this study received two cycles of HDCT is an important factor explaining this difference. Results of 364 patients who underwent a similar protocol from Indiana University were reported in 2017. Platinum refractory patients received direct HDCT, while platinum sensitive patients were treated with one or two cycles of standard dose VeIP followed by two consecutive 3-day high-dose CE protocols. Late relapse patients were not included. Two-year PFS was reported as 60% and 2-year OS as 66% [9]. A study by Feldman and colleagues at Memorial Sloan Kettering Cancer Center (MSKCC) investigated the outcomes of HDCT using the TI-CE regimen. The treatment consisted of two cycles of paclitaxel and ifosfamide, followed by ASCT and three consecutive cycles of HDCT-CE. The results showed a median PFS of 22 months and a 5-year OS rate of 52% [12]. Connolly et al. reported the results of HDCT collected from low-volume centers in Australia and New Zealand. Data from 111 patients were collected from 13 different centers. A total of 84% of the patients reported NSGCTs. Salvage CDCT was reported in 59% of the patients before HDCT. The 2- and 5-year PFS rates were 57% and 52%, and the OS rates were 65% and 61%, respectively [4]. More recently, long-term follow-up data of salvage four-course TIP treatment were reported. The 5-year PFS and OS rates were 66% and 69%, respectively, with a mean follow-up of 8.9 years. 27% of the patients had seminoma. There were 15% patients with high-risk IPFSG [13]. In our study, three cycles of TIP were applied, the proportion of IPFSG high and very high-risk patients was almost twice that of this study, and all of them were NSGCTs. While there is no study directly comparing TIP vs TIP + HDCT, it is important that the results of this study are similar to our study, but the patients in our study group have worse prognostic features. TIGER study is an international multicenter study that aims to determine whether CDCT or HDCT is the optimal first salvage chemotherapy approach in advanced GCTs patients. In this randomized controlled trial, patients with advanced disease who had progressed after initial treatment with cisplatin-based chemotherapy were assigned to either a control arm receiving CDCT with TIP or an experimental arm receiving HDCT with the TI-CE protocol. The primary objective of the study is to determine OS, while secondary endpoints include PFS, response rate, toxicity, quality of life, and biological markers. The results of this highly anticipated study are expected to establish whether HDCT or CDCT should be considered the standard of care for first-line salvage therapy [14]. HDCT is still one of the most important ways to achieve a cure for platinum-refractory disease.

Some of the patients in this study underwent resection of their residual lesions in the post-HDCT period. This approach can be preferred in all patients with refractory GCTs when appropriate. Lymph node dissection is an important type of resection here. Pelvic lymph node dissection is a difficult surgery with complications and management. Robotic surgery has become prominent in this field in recent years [15].

In most studies, platinum refractoriness is almost always included as a poor prognostic factor [3,5,8]. In our study, platinum refractoriness indicated a poor prognosis in terms of both PFS and OS. Many factors were analyzed as possible risk factors affecting survival. High serum hCG levels and AFP levels immediately before HDCT are also known to be variables affecting survival [3,5,8]. In the present study, we observed that platinum sensitivity and low hCG and AFP levels may have a positive effect on PFS and OS.

In recent years, it has been frequently studied that inflammation in the tumor microenvironment helps malignant cells proliferate and survive. This inflammation is thought to increase angiogenesis in the tumor microenvironment, facilitate metastases, and impair adaptive immunity. This situation has also been shown to play a role in the decreased response to chemotherapeutic agents [16]. Therefore, pretreatment evaluation of inflammation has become important in predicting the response to cancer treatment. The SII has gained attention as a potential prognostic marker in various types of cancer [17]. Fankhauser and colleagues investigated the predictive value of SII in patients with metastatic GCTs undergoing cisplatin-based chemotherapy. They also examined the neutrophil-to-lymphocyte ratio (NLR) separately, as well as in combination with SII. In a multivariate analysis, both NLR and SII emerged as independent predictors of OS [18]. Cursano and colleagues found that SII and NLR can forecast both PFS and OS in GCTs patients treated with HDCT. Their analysis also revealed a correlation between NLR and SII with the overall response to HDCT [19]. The fact that patients in this study received three consecutive cycles of HDCT is the biggest difference from our study. In our study, SII was significant in univariate and multivariable analyses for PFS, but not for OS. The lack of significance of SII on OS may be due to the treatments used in patients with refractory or relapsed course after HDCT. In post-HDCT use, treatments such as gemcitabine, paclitaxel and oxaliplatin (GemPOx) may have a significant contribution to survival [20].

Myelotoxicity due to HDCT can be managed with the use of G-CSF and appropriate antibiotics. Despite this, mortality may occur within the first 100 days after HDCT. According to the literature, most treatment-related mortality rates are around 10% or less, as in our study [21]. In this sense, we can say that one cycle HDCT after three cycles of TIP is a safe treatment approach. Due to the retrospective nature of our study, neuropathy and ototoxicity could not be evaluated. In most studies evaluating this treatment, these side effects were evaluated anecdotally. Beyer et al. reported neuropathy and ototoxicity in 29% and 18% of patients, respectively [22].

The median follow-up was calculated as approximately three years. This is a limiting factor in terms of evaluating long-term side effects. It is important to continue to follow these patients in this sense. Follow-up will be important in terms of maturing long-term survival data. Despite this, the high 2-year PFS and OS rates are important in terms of survival.

Our study faces several constraints worth noting. The cross-sectional nature of our analysis limits our ability to establish definitive cause-and-effect relationships between the variables examined. The retrospective design limits causal inferences. This limitation can be overcome by designing studies that prospectively evaluate such treatment approaches using conventional chemotherapy within the scope of reimbursement in resource-limited countries. As with many retrospective studies, there is an inherent risk of data inaccuracies. This is particularly problematic for complex parameters like platinum resistance, where precise determination can be challenging. Our analysis lacks comprehensive information on non-hematological side effects, such as neuropathy and hearing loss, due to insufficient documentation in patient records. The relatively brief follow-up period prevented us from evaluating the long-term effects of HDCT on patients’ quality of life and potential delayed side effects. These side effect parameters, which seriously affect the quality of life in this patient group where a long survival is expected despite relapse or refractory disease, should be recorded and evaluated in future studies. These limitations should be considered when interpreting our results.

## 5. Conclusions

The efficacy and safety of single HDCT treatment after three cycles of TIP after first relapse/refractory disease in young and advanced stage NSGCTs patients with high expectation of cure were demonstrated in this study. The existence of meaningful survival data for this patient group, for whom the standard of care is still unclear, will continue to affect daily practice at least until the results of the TIGER study are announced. Although three cycles TIP followed by HDCT seem to have significant data for this patient group, it cannot be ignored that this treatment preference emerged as a result of healthcare infrastructure. It is a fact that sequential HDCT applications in global centers are the standard of care if HDCT is to be preferred. In terms of optimal HDCT use in low-volume or resource-limited settings, HDCT may be preferred after salvage chemotherapy. Maintenance single-agent metronomic therapies may be added in the post-HDCT period. There is also a need for randomized controlled trials to compare TIP + HDCT with other salvage therapies like four cycles of TIP or other triplet regimens. In these studies, biomarkers other than tumor markers that will predict response to treatment and prognosis are now needed. In this sense, studies on the use of miRNA can be moved from evaluation in early-stage disease to salvage period [23].

## Figures and Tables

**Table 1:** Clinical characteristics of patients before HDCT and survival parameters after HDCT.

Characteristics	Total (*n* = 141)
**Gender, male, *n* (%)**	141 (%100)
**Primary, gonadal, *n* (%)**	141 (100)
**Age, years, median (min–max)**	27 (18–51)
**Age, <30 years, *n* (%)**	86 (61)
**Histology, Non-seminoma, *n* (%)**	141 (100)
**Non-seminoma subtype, *n* (%)**	
- Mixed Germ Cell	108 (76.6)
- Choriocarcinoma	4 (2.8)
- Embryonal Carcinoma	16 (11.3)
- Yolk Sac	5 (3.5)
- Immature Teratoma	8 (5.7)
**Clinical stage at first diagnosis, (AJCC, 8th), *n* (%)**	
- Stage I–II	50 (35.5)
- Stage III	91 (64.5)
**IGCCCG, *n* (%)**	
- Good Risk	35 (24.8)
- Intermediate Risk	23 (16.3)
- Poor Risk	83 (58.9)
**History of first line chemotherapy, *n* (%)**	
- BEP/EP	138 (97.9)
- VIP	3 (2.1)
**Metastatic areas before HDC, *n* (%)**	
- Lung	98 (69.5)
- Lymph nodes	130 (92.2)
- Liver	27 (19.1)
- Bone	15 (10.6)
- Brain	18 (12.8)
**Liver, Bone, Brain metastasis before HDC, *n* (%)**	
- Present	49 (34.8)
**Platinum Refractory Disease, *n* (%)**	
- Present	6 (4.3)
**Late Relapse, *n* (%)**	
- Present	20 (14.2)
**AFP before HDC, *n* (%)**	
- ≥9 ng/mL	29 (20.6)
**Beta HCG before HDC, *n* (%)**	
- ≥1 mIU/mL	36 (25.5)
**AFP and/or Beta HCG elevation before HDC**	
- Present	58 (41.1)
**IPFSG, *n* (%)**	
- Low risk	26 (18.4)
- Intermediate risk	74 (52.5)
- High risk	25 (17.7)
- Very high risk	16 (11.3)
**HDC protocol, *n* (%)**	
- CE	135 (95.7)
- ICE	6 (4.3)
**Follow up (months), median (SE)**	35.2 (2.9)
**Response after HDC** **, *n* (%)**	
- CR or marker negative PR	84 (59.6)
- Marker positive PR or SD	24 (17)
- PD	33 (23.4)
- Disease control rate	108 (76.6)
**Progression Free Survival, median (IQR)**	NR (NR-NR)
**2 year PFS rate, % (SE)**	51.8 (4.4)
**Overall Survival, median (IQR)**	NR (NR-NR)
**2 year OS rate, % (SE)**	72.3 (4)
**Resection after HDC, *n* (%)**	
- Present	20 (14.2)
SII *	
- Low	73 (51.8)
- High	68 (48.2)

* The optimal SII threshold value was calculated as 610.18 in ROC analyses. This SII value had 59.5% sensitivity and 59.7% specificity for 24-month PFS prediction. (AUC 0.642; 95% CI 0.551–0.733; *p* = 0.004). Abbreviations: AJCC: American Joint Committee on Cancer, IGCCCG: International Germ Cell Cancer Collaborative Group, HDC: High Dose Chemotherapy, IPFSG: International Prognostic Factors Study Group, BEP/EP: Bleomycin, etoposide, and cisplatin/etoposide and cisplatin, VIP: Etoposide, ifosfamide, and cisplatin, AFP: Alpha Fetoprotein, HCG: Human Chorionic Gonadotropin, CR: Complete response, PR: Partial response, SD: Stabile Disease, PD: Progressive Disease, SE: Standard Error, IQR: Interquartile range, PFS: Progression free survival, OS: Overall survival, SII: Systemic immune-inflammation index.

**Table 2:** Side effects parameters of patients after HDCT.

Characteristics	Total (*n* = 141)
**Engraftment Day, (median) IQR**	12 (3)
**Neutropenia, *n* (%)**	
- Grade 4	141 (100)
**Anemia, *n* (%)**	
- Absent	1 (0.7)
- Grade 1	36 (25.4)
- Grade 2	94 (66.7)
- Grade 3	9 (6.4)
- Grade 4	1 (0.7)
**Thrombocytopenia, *n* (%)**	
- Grade 1	2 (1.4)
- Grade 2	0 (0)
- Grade 3	3 (2.1)
- Grade 4	136 (96.5)
**Number of ES transfusions after HDC, median (IQR)**	2 (2)
**Number of TS transfusions after HDC, median (IQR)**	4 (4)
**Kidney Failure, *n* (%)**	
- Need for hemodialysis	3 (2.1)
**Liver enzyme elevation, *n* (%)**	
- Absent	91 (64.5)
- Grade 1	26 (18.4)
- Grade 2	15 (10.6)
- Grade 3	7 (5)
- Grade 4	2 (1.4)
**Transplantation-related mortality, *n* (%)**	
- Present	10 (7.1)

Abbreviations: IQR: Interquartile range, ES: Erythrocyte Suspension, TS: Thrombocyte suspension, HDC: High Dose Chemotherapy.

**Table 3:** Univariate and multivariable analysis of progression free survival.

Variables	*n* (%)	Median PFS, month (95% CI)	Univariable *p*-Value	Multivariable *p*-Value	HR (95% CI)
**Age**					
<30	86 (61)	NR	0.077		
≥30	55 (39)	10.7 (5.5–15.9)			
**Histology, Non-seminoma**					
Mixt Germ cell tumors	108 (76.6)	NR	**0.032**	0.093	NE
Coryocarcinoma	4 (2.8)	8 (2.3–13.8)		0.657	0.2–2.74
Embriyonal carcinoma	16 (11.3)	NR		0.568	0.52–3.2
Yolk sac tumor	5 (3.5)	3.5 (1.4–5.5)		**0.011**	**1.34–9.9**
Immature teratoma	8 (5.7)	NR		0.327	0.15–1.86
**Clinical stage at first diagnosis, (AJCC, 8th)**					
Stage I–II	50 (35.5)	NR	0.142		
Stage III	91 (64.5)	15.8 (-)			
**IGCCCG**					
Good	35 (24.8)	NR	**0.046**	0.79	NE
Intermediate	23 (16.3)	NR		0.71	0.31–2.21
Poor	83 (58.9)	11.8 (3.6–20)		0.72	0.57–2.2
**Pulmonary metastasis**					
Yes	43 (30.5)	NR	**0.021**	0.15	0.84–2.96
No	98 (69.5)	11.8 (0–27.6)			
**Liver, Bone, Brain metastasis**					
Yes	49 (34.8)	NR	**0.020**	0.092	0.92–3
No	92 (65.2)	7.5 (3.2–11.7)			
**Platinum Refractory Disease**					
Yes	6 (4.3)	NR	**0.001**	**0.021**	1.22–11.2
No	135 (95.7)	3 (2.4–3.5)			
**Late Relapse**					
Absent	121 (85.8)	NR	0.133		
Present	20 (14.2)	11.7 (5.8–17.5)			
**IPFSG**					
Low risk	26 (18.4)	NR	**0.001**	0.976	
Intermediate risk	74 (52.5)	NR			
High risk	25 (17.7)	11.8 (0–30.9)			
Very high risk	16 (11.3)	3.9 (1.35–6.4)			
**AFP and/or Beta HCG elevation before HDC**					
Absent	83 (58.9)	NR	**0.001**	**0.001**	2.9–9.6
Present	58 (41.1)	6.23 (3.3–9.0)			
**HDC protocol, *n* (%)**					
CE	135 (95.7)	NR	0.444		
ICE	6 (4.3)	10.4 (0.1–20.7)			
**Response after HDC**					
CR or marker negative PR	84 (59.6)	NR	0.001		
Marker positive PR or SD	24 (17)	7.5 (4.4–10.5)			
PD	33 (23.4)	2.6 (1.7–3.6)			
**Resection after HDC**					
Absent	121 (85.8)	NR			
Present	20 (14.2)	27.66 (0.17–55.1)	0.754		
**SII**					
Low	73 (51.8)	NR	**0.026**	**0.027**	1–3.2
High	68 (48.2)	10 (5.9–14)			

**Abbreviations:** NR: Not Reached, AJCC: American Joint Committee on Cancer, IGCCCG: International Germ Cell Cancer Collaborative Group, HDC: High Dose Chemotherapy, IPFSG: International Prognostic Factors Study Group, AFP: Alpha Fetoprotein, HCG: Human Chorionic Gonadotropin, CR: Complete response, PR: Partial response, SD: Stabile Disease, PD: Progressive Disease, PFS: Progression free survival, SII: Systemic immune-inflammation index.

**Table 4:** Univariate and multivariable analysis of overall survival.

Variables	*n* (%)	Median OS, month (95% CI)	Univariable *p*-Value	Multivariable *p*-Value	HR (95% CI)
**Age**					
<30	86 (61)	NR	0.295		
≥30	55 (39)	NR			
**Histology, Non-seminoma**					
Mixt Germ cell tumors	108 (76.6)	NE	**0.046**	0.555	NE
Coryocarcinoma	4 (2.8)	NE		0.663	0.33–5.6
Embriyonal carcinoma	16 (11.3)	NE		0.466	0.44–5.7
Yolk sac tumor	5 (3.5)	NE		0.094	0.82–12.3
Immature teratoma	8 (5.7)	NE		0.979	NE
**Clinical stage at first diagnosis, (AJCC, 8th)**					
Stage I–II	50 (35.5)	NR	0.143		
Stage III	91 (64.5)	NR			
**IGCCCG**					
Good	35 (24.8)	NR	**0.013**	0.223	NE
Intermediate	23 (16.3)	NR		0.867	0.24–5.4
Poor	83 (58.9)	NR		0.131	0.78–6.5
**Pulmonary metastasis**					
Yes	43 (30.5)	NR	**0.042**	0.298	0.66–3.8
No	98 (69.5)	NR			
**Liver, Bone, Brain metastasis**					
Yes	49 (34.8)	NR	**0.014**	0.852	0.5–2.27
No	92 (65.2)	NR			
**Platinum Refractory Disease**					
Yes	6 (4.3)	6.56 (3.7–9.4)	**0.001**	**0.030**	1.14–13.21
No	135 (95.7)	NR			
**Late Relapse**					
Absent	121 (85.8)	NR	0.750		
Present	20 (14.2)	NR			
**IPFSG**					
Low risk	26 (18.4)	NR	**0.001**	**0.002**	**NE**
Intermediate risk	74 (52.5)	NR		0.140	0.6–37.03
High risk	25 (17.7)	NR		0.278	0.37–30.54
Very high risk	16 (11.3)	7.33 (1.06–13.6)		**0.010**	**1.96–150.53**
**AFP and/or Beta HCG elevation before HDC**					
Absent	83 (58.9)	NR	**0.001**	**0.001**	2.36–11.84
Present	58 (41.1)	17.06 (0–38.1)			
**HDC protocol, *n* (%)**					
CE	135 (95.7)	NR	0.844		
ICE	6 (4.3)	NR			
**Response after HDC**					
CR or marker negative PR	84 (59.6)	NR	**0.001**		
Marker positive PR or SD	24 (17)	NR			
PD	33 (23.4)	6.16 (4.04–8.2)			
**Resection after HDC**					
Absent	121 (85.8)	NR	0.122		
Present	20 (14.2)	NR			
**SII**					
Low	73 (51.8)	NR	0.063		
High	68 (48.2)	NR			

**Abbreviations:** NE: Not Evaluable. NR: Not Reached, AJCC: American Joint Committee on Cancer, IGCCCG: International Germ Cell Cancer Collaborative Group, HDC: High Dose Chemotherapy, IPFSG: International Prognostic Factors Study Group, AFP: Alpha Fetoprotein, HCG: Human Chorionic Gonadotropin, CR: Complete response, PR: Partial response, SD: Stabile Disease, PD: Progressive Disease, OS: Overall survival, SII: Systemic immune-inflammation index.

## Data Availability

The original contributions presented in the study are included in the article; further inquiries can be directed to the corresponding author.
